# Parkin Controls Cardiac Function in Obesity by Regulating Mitochondrial Calcium Uptake∗

**DOI:** 10.1016/j.jacbts.2022.04.015

**Published:** 2022-08-22

**Authors:** Stanislovas Jankauskas, Urna Kansakar, Antonio De Donato, Pasquale Mone, Fahimeh Varzideh, Gaetano Santulli

**Affiliations:** a Department of Medicine (Cardiology), Wilf Family Cardiovascular Research Institute, Einstein Institute for Aging Research, Einstein Institute for Neuroimmunology and Inflammation, Albert Einstein College of Medicine, New York, New York, USA; b Department of Translational Medical Sciences, Genetic Research Institute “Gaetano Salvatore,” University of Campania “Luigi Vanvitelli,” Naples, Italy; c Department of Molecular Pharmacology, Fleischer Institute for Diabetes and Metabolism, Einstein-Sinai Diabetes Research Center, Albert Einstein College of Medicine, New York, New York, USA

**Keywords:** Ca^2+^ overload, high-fat diet, mitochondria, Parkin, VDAC1

**Figure:**
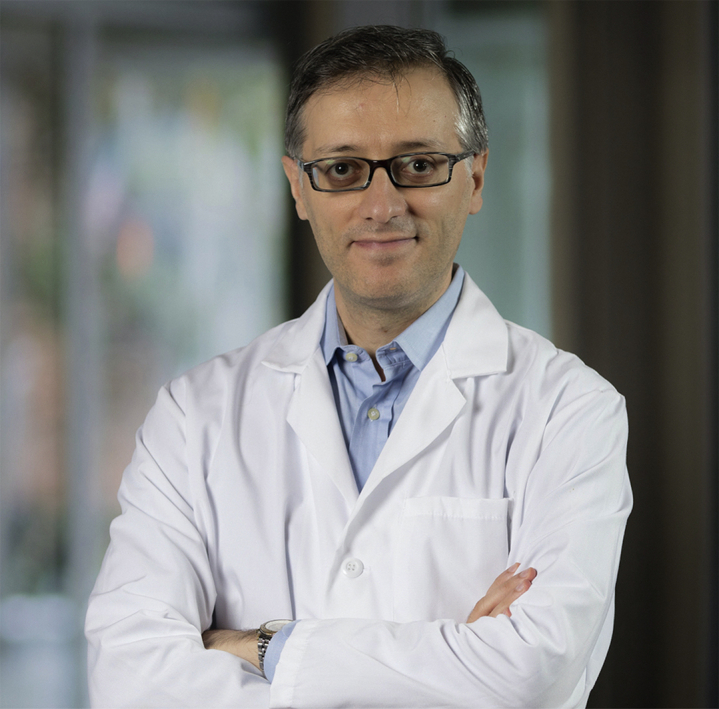

The global prevalence of obesity nearly tripled between 1975 and 2016, making it one of the current biggest challenges for health care. In the United States, obesity affects about 40% of the adult population, and a substantial increase in obesity prevalence has been detected in low-income and middle-income nations. High values of body mass index are associated with numerous health problems, including but not limited to atherosclerosis, coronary heart disease, insulin resistance, hyperglycemia, nonalcoholic fatty liver disease, iron-deficiency anemia, and chronic inflammation. Radical changes in lifestyle and diet habits have been shown to significantly reduce the risk for complications and disease development in obese patients. Unfortunately, these changes are not easy to achieve for many patients.

The perplexing entanglement of the underlying mechanisms makes obesity one of the most complicated and intriguing research topics. Thus, research on the mechanisms of heart failure in obesity represents a vital topic.

Obesity triggers a hard bioenergetic challenge for the heart, which initially responds with left ventricular hypertrophy. However, the decreased contractile efficacy of the myocardium pushes the heart to promote hypertrophy further, eventually resulting in heart failure.^1^ Reduced contractility relates to impaired intracellular calcium handling on one hand and to disrupted cardiomyocyte bioenergetics on the other hand. Obesity elicits numerous pathologic changes that affect myocardial bioenergetics on the anatomical, physiological, cellular, and biochemical levels. Mitochondria are crucial for heart bioenergetics, being the ultimate target of all these pathologic alterations.^1^ The study performed by the research team led by Jun Ren and published in this issue of *JACC: Basic to Translational Science*^2^ is of special interest, as it aimed at connecting the alterations in cardiomyocyte bioenergetics found in obesity with dysregulated calcium handling. The research specifically focused on elucidating the connection between mitophagy and calcium handling.

What is the relationship between mitochondrial calcium and mitophagy? Mitophagy plays an instrumental role in myocardial health in obesity, enabling the elimination of mitochondria that are somehow inefficient in producing adenosine triphosphate (ATP). This task is achieved through a Parkin–PTEN-induced kinase 1 (PINK1) mechanism, in which PINK1 accumulates on the outer mitochondrial membrane of mitochondria with low transmembrane potential. ATP production rate directly correlates with mitochondrial potential; thus PINK1 accumulation marks bioenergetically inefficient mitochondria. At the same time, PINK1 activates Parkin, an E3 ubiquitin ligase. Hence, the Parkin-PINK1 system makes it possible to distinguish “healthy” mitochondria from “unhealthy,” bioenergetically inefficient organelles.^3^

Cardiac Parkin expression is down-regulated in obesity, as elegantly established in human heart samples, a murine in vivo model (high-fat diet [HFD]), and in vitro assays (isolated adult murine cardiomyocytes treated with palmitic acid). Moreover, the inhibition of cardiac mitophagy in obesity was confirmed using all 3 models. A Parkin-knockout mouse was harnessed to find out the exact role of Parkin down-regulation in these settings. An HFD elicited a more profound alteration in cardiac function in Parkin-knockout mice compared with wild-type mice, substantiating that Parkin-mediated cardiac mitophagy plays a protective role in obesity. Intriguingly, obesity-induced down-regulation of Parkin attenuated both the increase in cytoplasmic calcium during contraction and the speed of its reuptake in the diastolic phase.

One of the most fascinating parts of the study is the mechanistic identification of a molecular culprit. Indeed, 2 main targets of Parkin are voltage-dependent anion-selective channel 1 (VDAC1) and mitochondrial calcium uniporter (MCU), which transport calcium through the outer and inner mitochondrial membranes, respectively (Figure 1). Being a E3 ligase, Parkin is able to ubiquitinate several proteins, including the main mitochondrial calcium import proteins. Thus, the down-regulation of Parkin mediated by fatty acids causes increased accumulation of VDAC1 and MCU, whereas the overexpression of Parkin reduces their abundancy. Moreover, consistent with previous findings in heart failure,^4^ mitochondrial calcium overload was detected in cardiomyocytes from HFD mice, and it was further exacerbated by Parkin knockout.

**Figure 1:**
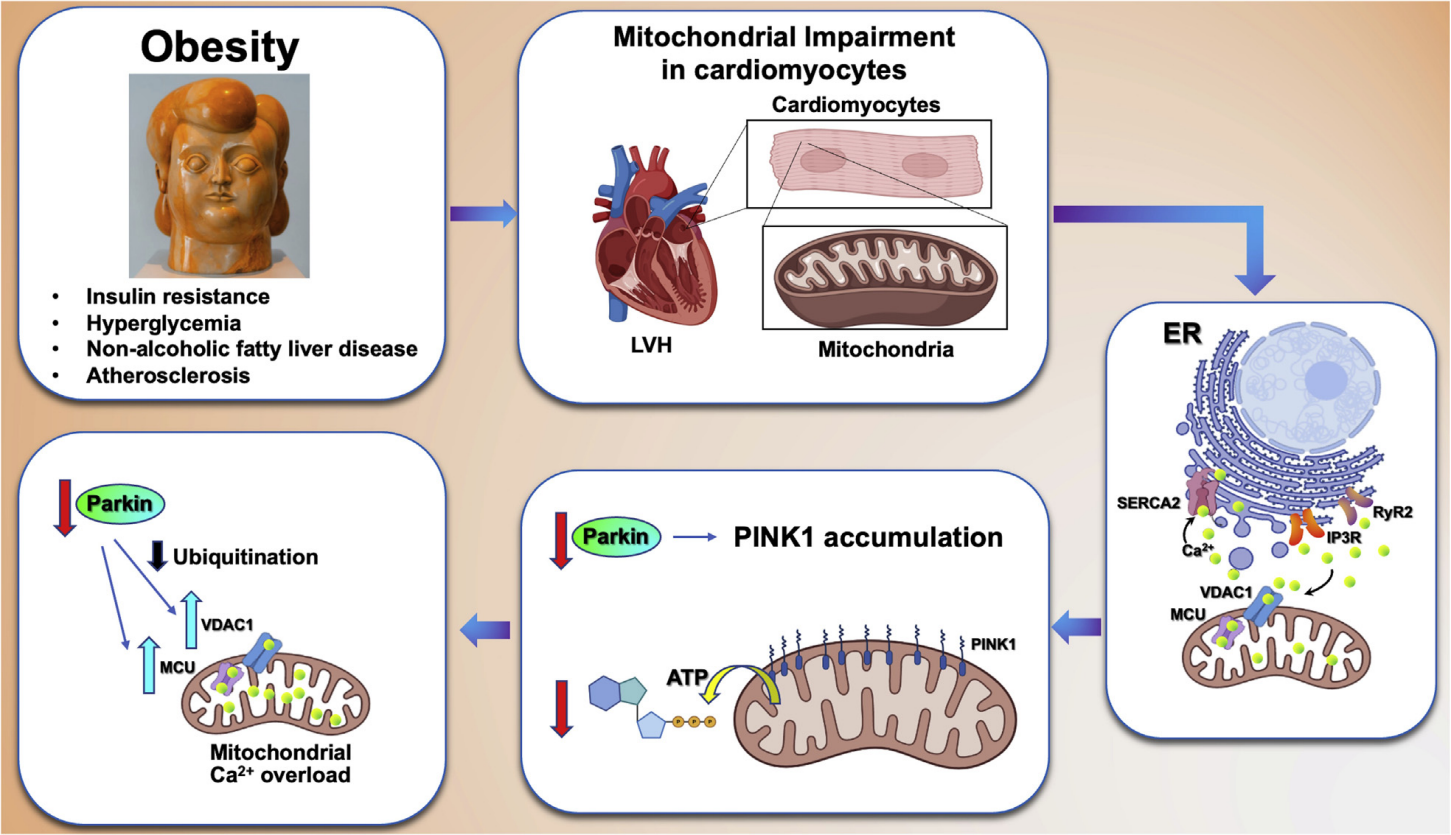
Schematic Representation of the Molecular Mechanisms Linking Parkin and Cardiac Mitochondrial Calcium Overload in Obesity ER = endoplasmic reticulum; IP3R = inositol 1,4,5 trisphosphate receptor; LVH = left ventricular hypertrophy; MCU = mitochondrial calcium uniporter; PINK1 = PTEN-induced kinase 1; RyR2 = ryanodine receptor; SERCA2 = sarco/endoplasmic reticulum calcium–ATPase 2; VDAC1 = voltage-dependent anion-selective channel 1.

These findings are very thought provoking, as they clearly demonstrate that mitophagy in cardiomyocytes is critical not only as a goalkeeper of mitochondrial ATP production but also as a fine orchestrator of calcium handling and thus cardiomyocyte contraction.

Strengths of the study include replication of the main findings in left ventricular samples obtained from unsuccessful cardiac transplants from lean and obese patients, providing major translational implications. Nonetheless, this work has some limitations, such as the use of a global knockout mouse instead of a cardiomyocyte-specific one, and it leaves a number of open questions, including the functional role of PINK1 in the described processes and the potential molecular mechanisms linking mitophagy and autophagy with the effects of obesity on cardiac transcriptomics and metabolomics. Further investigations in this sense may increase the clinical relevance of this discovery, as now we do not possess any reliable pharmacologic tools for the up-regulation of Parkin expression. The connection between calcium overaccumulation in mitochondria and disruption of calcium handling also leaves some stimulating knowledge gaps. For instance, mitochondrial calcium uptake is described to rely massively on the interconnection between endoplasmic reticulum (ER) and mitochondria. Disruption of ER-mitochondrial communications decreases mitochondrial calcium uptake, and a loss of ER-mitochondrial contacts has been reported in obesity.^5^

So, is mitochondrial calcium good or bad? Mitochondrial calcium overload detected in Parkin-knockout cardiomyocytes does not seem to reconcile with the reduced calcium reuptake observed in the same setting. Most likely these events are caused by reduced activity of sarco/endoplasmic reticulum calcium–ATPase 2 (SERCA2), attributable to a decrease in ATP generation. The latter connects the alteration in bioenergetics to dysfunctional excitation-contraction coupling. However, mitochondrial energy production in absence of Parkin was not assessed. This aspect is one of the main limitations of the study, as calcium plays a double role in mitochondrial function. Indeed, whereas low calcium concentrations reduce oxidative phosphorylation, high calcium concentrations prompt the opening of the mitochondrial permeability transition pore, eventually resulting in a collapse of mitochondrial potential and ATP production. Although some indirect evidence for mitochondrial permeability transition pore opening is provided by the increased expression of VDAC1 and MCU with an HFD, this finding is not sufficient though to definitively dissect the bioenergetic effects of mitochondrial calcium uptake in these conditions. Therefore, mitochondrial calcium needs to stay at a “*lagom*” level: *Lagom* is a Swedish term that summarizes in a single word the concept of not too much, not too little, just right, which exquisitely applies to the physiology of mitochondrial calcium.

## Funding Support and Author Disclosures

Dr Santulli’s laboratory is supported in part by the National Institutes of Health (grants R01-HL159062, R01-DK123259, R01-DK033823, R01-HL146691, and T32-HL144456 to Dr Santulli), by the Irma T. Hirschl and Monique Weill-Caulier Trusts, and by the Diabetes Action Research and Education Foundation. Drs Jankauskas and Varzideh hold postdoctoral fellowships from the American Heart Association (AHA-21POST836407 and AHA-22POST915561, respectively). The authors have reported that they have no relationships relevant to the contents of this paper to disclose.
